# Bio-inspired Surface Texture Modification as a Viable Feature of Future Aquatic Antifouling Strategies: A Review

**DOI:** 10.3390/ijms21145063

**Published:** 2020-07-17

**Authors:** Chloe Richards, Asma Slaimi, Noel E. O’Connor, Alan Barrett, Sandra Kwiatkowska, Fiona Regan

**Affiliations:** 1 DCU Water Institute, School of Chemical Sciences, Dublin City University, Glasnevin, Dublin 9, Ireland; chloe.richards3@mail.dcu.ie (C.R.); alan.barrett9@mail.dcu.ie (A.B.); sandra.kwiatkowska2@mail.dcu.ie (S.K.); 2 Insight Centre for Data Analytics, Dublin City University, Dublin 9, Ireland; asma.slaimi@insight-centre.org (A.S.); noel.oconnor@insight-centre.org (N.E.O.)

**Keywords:** biofouling, marine inspiration, topography, surface modification, antifouling

## Abstract

The imitation of natural systems to produce effective antifouling materials is often referred to as “biomimetics”. The world of biomimetics is a multidisciplinary one, needing careful understanding of “biological structures”, processes and principles of various organisms found in nature and based on this, designing nanodevices and nanomaterials that are of commercial interest to industry. Looking to the marine environment for bioinspired surfaces offers researchers a wealth of topographies to explore. Particular attention has been given to the evaluation of textures based on marine organisms tested in either the laboratory or the field. The findings of the review relate to the numbers of studies on textured surfaces demonstrating antifouling potential which are significant. However, many of these are only tested in the laboratory, where it is acknowledged a very different response to fouling is observed.

## 1. Introduction

Biofouling is a major problem in marine waters where most immersed surfaces become fouled to some extent, developing large amounts of biomass. Advanced biofouling in marine waters can often accumulate such significant biomass that biofouling is often further subdivided into two subdivisions; microfouling and macrofouling. Microfouling is a type of fouling composed of microbial organisms such as bacteria and diatoms. Macrofouling is caused by the accumulation of larger life forms such as barnacles, bryozoans, polychaetes and macro-algae [1,2]. Microfouling is considered a necessary precursor to the development of a macrofouling community and can be detrimental to the deployment of sensitive equipment such as environmental sensors over time scales of just two weeks in areas of high fouling pressure [3]. A search for new non-toxic marine coatings meant that the opportunity to explore “green” methods of antifouling had arisen, with the consequence that developing non-biocidal methods of preventing fouling received much attention where natural fouling defense mechanisms have been mimicked through chemical, physical, and/or stimuli-responsive methodologies [4]. In 2010 the final report of a 60-month European project: Advanced nanostructured surfaces for the control of biofouling (AMBIO) was published [5]. The team of scientists reported the study of 500 different nanostructured coatings, representing 64 generic coating chemistries that were prepared at laboratory-scale and evaluated for their antifouling and fouling-release performance. The study led to fifteen coatings selected for testing in a range of field and end-user scenarios. Several coatings showed promise, some leading to a commercialized product and others showing potential for further development [6,7,8,9,10,11,12,13,14].

**Figure 1:**
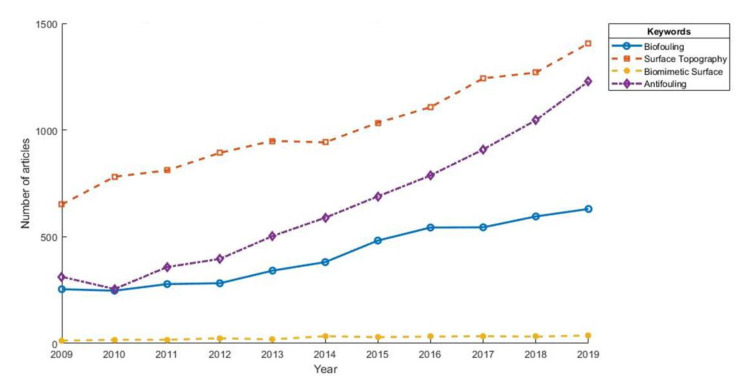
Comparison of the research trends of keyword selected articles from 2009–2019 indexed by Web of Science.

Figure 1, below, shows the comparison of the growth trends looking at five key research subject areas from 2009–2019 indexed by Web of Science. Surprisingly, the subject area of biomimetic surfaces was among the least researched areas of interest during the past 10 years. This review intends to highlight some of the key contributions made in the development of antifouling solutions based solely on textured surfaces inspired by the marine environment, whilst offering some insight into those areas that need further exploration.

Biofouling is a surface-based phenomenon therefore it is not surprising that the substrate-environment interface has a significant influence on the type, rate and extent of fouling that may occur [8,9,10,11,12]. A range of fabrication techniques is available to produce a wide variety of designed surface structures with high fidelity and relatively low-cost compared to previous decades [15]. There are extensive examples in the literature of sophisticated submicrometer scale pattern fabrication [14,16,17]. Previously, surface features were categorized as either structural topography or chemical patterning, however at the nanoscale, the distinction between purely physical and purely chemical patterning of surfaces is now being eroded. Both 3-D physical and chemical nanoscale organization are now possible within a range of methods, including but not limited to; optical lithography, microcontact printing, electron beam lithography, ion beam lithography, soft lithography, direct laser interference patterning and 3-D printing. The knowledge of nanotechnology, and with it, the ability to manipulate surface nanostructure, offers the potential to both enhance the efficacy of existing materials and to produce a completely novel, and perhaps a non-toxic mechanism of antimicrobial activity [12,18,19]. The aim of this review is to show the earliest and latest knowledge, surrounding biological responses to marine inspired surface topography. It deals with the potential of surface modification in general, and techniques used, as a viable component of future aquatic antifouling strategies.

## 2. Surface Modification

The study of surface topographical features has become increasingly popular, with numerous studies reporting intricate natural topographies found on many organisms that are known to resist fouling. The replication of artificial surfaces inspired by nature has produced many promising results [15,20,21,22,23]. Many studies have shown a mixture of attachment, depending on the size and shape of the organism and the specific microtexture used as a fouling-resistant mechanism. However, the explanation behind this attachment is still not well understood. A number of theoretical models have been proposed over the years to explain this attachment. One of these models is the Derjaguin–Landau–Verwey–Overbeek (DLVO) theory [24,25,26]. The DLVO theory is expressed as shown in Equation (1):

∆*G^adh^* (*d*) = ∆*G^υdW^* (*d*) + Δ*G^dl^* (*d*) (1)

where ∆*G^adh^* refers to the attractive van der Waals forces, ∆*G^dl^* to the electrostatic repulsion forces and ∆*G^adh^* to the sum of the interaction of bacteria with a substrate [27,28,29].

The foundation of the DLVO theory is to differentiate interactions between colloidal particles or a colloidal particle and a substrate. This theory also offers an explanation behind the adhesion of algal cells to a surface. Bacteria cells range in size from 0.5–2 µm, similar to the size of colloidal particles; this theory has been extensively used in material science to explain the interactions that occur between bacteria and a substrate. Equation (1) above shows that both the van del Waals forces, ΔG^υdW^, and the electrostatic repulsion forces, ΔG^dl^, are dependent on the distance between a cell and a surface. A later extension of this theory led to a theoretical model called the extended DLVO theory. This model accounts for hydrophobic, hydrophilic and osmotic interactions (although osmotic interactions were later said to have little effect in bacterial adhesion) [27,28,29]. The extended DLVO theory can be summarized in the Equation (2):

∆*G^adh^* (*d*) = ∆*G^υdW^* (*d*) + Δ*G^dl^* + Δ*G^ab^* (2)

where ∆*G^ab^* refers to acid and base interactions [28].

Another theoretical model proposed for the explanation behind cell adhesion is that of thermodynamic theory. Thermodynamic theory expresses forces (i.e., van der Waals, electrostatic and dipole) on the basis of free energy [28]. Thermodynamic theory can be summarized by Equation (3):

∆*G^adh^* = *γ_sm_* − *γ_sl_* − *γ_ml_* (3)

where *γ_sm_* refers to the solid-microorganism, *γ_sl_* to the solid-liquid and *γ_ml_* to the microorganism-liquid free energies [28].

The basis of the thermodynamic theory relies on free energy; essentially, adhesion will occur on a surface if the free energy is negative. Unlike the conventional DLVO theory, thermodynamic theory does not care about the distance between the cell and its substrate. This theory assumes that bacterial interaction with a substrate is reversible, which may not always be the case—it does not explain the behavior observed in bacterial systems. However, it does state common observations in relation to wettability; hydrophilic surfaces will attract bacteria with hydrophilic properties and hydrophobic surfaces will attract bacteria with hydrophobic properties [24,25].

A popular mechanism used to explain the adhesion of cells to a substrate is attachment point theory [30,31]. Here, the fouling organism experiences increased attachment where there are multiple attachment points and reduced attachment when the number of attachment points are decreased. This can often be related to microtexture in the sense that highly complex topographies (i.e., whereby the microtexture is smaller than that of the organism) will not be favorable for attachment. On the other hand, where the microtexture is larger than the organism, settlement is reported to occur [32]. The work of Lorenzetti et al. confirms previously cited examples about the correlation between bacterial adhesion and a substrate [33].

Over the past number of years, developments in technologies to produce surface topographies at the micro- to nano-scale level have grown tremendously and allowed for numerous “cell-surface interaction studies” [34]. Many different surface topographies at both the micro- and nano-scale level (i.e., channels, pillars, riblets, pits) were obtained through the use of various different fabrication methods [34].

### 2.1. Production Methods

In order to be able to assess the organism-surface interaction, it is often necessary to replicate these surfaces for testing purposes. The work of Jinhong Fu and coworkers and Marin Steenackers and coworkers as part of the AMBIO project, has shown the development of hierarchical structures on surfaces [27,28]. Fu et al. [35] defined a controllable way to produce hierarchical micro- and nanostructured surfaces simultaneously by changing the pH. This enabled the tuning of the size range of the morphologies. The topography of the multilayer structure was fixed by thermal cross-linking and turned into a superhydrophobic surface by the chemical vapor deposition of (tridecafluoroctyl)-triethoxysilane. In a separate study by Steenackers et al. [36], self-initiated photografting and photopolymerization (SIPGP) of styrene and acrylic monomers on structured ω-functionalized biphenylthiols self-assembled monolayers (SAMs) on gold was shown. This was a three-step approach allowing the preparation of defined structured polymer brushes without the need of a specific surface bonded photoinitiator function. Polymer brushes were selectively formed on cross-linked SAM regions. The polymer layer thickness was controlled by the extent of electron-induced cross-linking and head group conversion of the SAM layer [5,28]. In a recent study, picosecond (ps) laser texturing of stainless steel was carried out by Sun et al. [37], generating micro-groove and micro-pit arrays which were tested in the laboratory in artificial seawater. The results were reported to be a fast, highly controllable picosecond laser patterning way for preparing hierarchical micro/nanostructures, combined with the chemical modification by silica sol, was proposed to fabricate the anti-biofouling stainless steel superhydrophobic surfaces (SHSs). The results of five weeks seawater immersion test showed that the specimens with SHS demonstrate significant anti-biofouling effect. It is not clear if the texture alone can provide valuable inhibition of biofouling—though the technique is worth considering due to its applicability to steel.

In addition to the elegant chemical methods of generating surface features and topographies, the growing interest in materials science, has led to a wide range of physical fabrication methods. These methods are used to replicate and/or produce textures that are inspired by either attachment point theory or by surface features of natural organisms. These are summarized in Table 1.

Controlling cellular interaction with a surface is often complex, demanding careful consideration of multiple factors such as roughness, wettability, hydrodynamics, mechanical properties and topography. Many fouling organisms (i.e., bacteria, diatoms) exist in the micrometer size range with nanometer size ranges of surface attributes. Learning to control surface topography in these micro- and nano-scale levels plays a crucial role in understanding and thus, controlling bacterial attachment and biofilm formation [38].

### 2.2. Surface Roughness

Early indications show that substrate roughness and topography increase the adhesion of most common fouling groups, and this is attributed to features allowing protection from hydrodynamic shear forces of removal and predation, or by increasing the surface area available for attachment. Most early studies that considered the influence of substrate roughness on fouling accumulation typically did not report any attempts to characterize the roughness scales [63]. However, the later re-evaluation of surface roughness has indicated that rather than a function of the purely passive mechanisms, active exploration of suitable surfaces for settlement leads to increased settlement on “preferred” surfaces [64]. Table 2 details the scale lengths of surface topographies commonly found on developed antifouling materials.

### 2.3. Surface Wettability

Surface wettability and surface energy are important characteristics of a material in both nature and in technology development. How the nature of the topography of a given surface influences the wettability of that surface, particularly in terms of establishing (super)hydrophobic surfaces, is now well established. It is now accepted that superhydrophobicity can only be obtained by introducing a certain degree of surface roughness, that is, a low surface energy is not enough [66,67,68,69], a surface’s wettability is defined by its water contact angle (WCA), described by Young in 1804 [69]. It is accepted that when the contact angle is <90° the surface is hydrophilic; when the contact angle ≥90° the surface is hydrophobic. A surface having a water contact angle ≥150° is usually classified as superhydrophobic, i.e., water repellent. Young determined that the equilibrium contact angle, θ_o_ of a liquid droplet on a flat substrate is determined by the interfacial energies, between the substrate, the liquid and its vapor (Equation (4).

cos*θ_o_* = (*γ_sv_* − *γ_sl_*/*γ_ml_*) (4)

The hydrophobicity of a smooth surface is limited by the surface’s chemistry; however the wetting behavior of a surface is also dependent on a surface’s topography [70,71]. Surface roughness can have a dramatic impact on the materials hydrophobicity/hydrophilicity. This effect of roughness on the contact angle was first considered by Wenzel [71]. He recognized the importance of surface roughness and proposed a modification to Young’s equation, which included a roughness factor, r, defined as the ratio between the actual rough surface area and the geometric projected area. According to Wenzel’s equation, a solid substrate with wetting tendency (θ < 90°) will wet more easily if its surface is rough, but, on the other hand a solid substrate with water repelling tendency (θ > 90°) will repel more when having a rough surface (Equation (5).

cos*θ_r_^w^* = *r* cos*θ_o_* (5)

However Young and Wenzel only considered chemically homogeneous surfaces. Cassie and Baxter [70] extended Wenzel’s work to non-homogeneous and porous surfaces. Cassie and Baxter equations can be also applied to rough hydrophobic surfaces. Equation (6) shows that as the surface is considered as a composite of solid and air, with a contact angle of θ_r^c:

cos*θ_r_^c^*= *f*(cos*θ_o_* + 1) − 1 (6)

where f is the fraction of liquid—solid contact, the composite contact is established when θ_o_ > θ_c_ and the threshold contact angle is defined by: cos*θ_c_* = *(f − 1)/(r − f).* So, for a hydrophobic rough surface, the liquid repellency prevents the liquid from fully penetrating into the depressions of the roughness morphology. Penetration of pores will occur spontaneously only for θ < 90° [72,73]. From a self-cleaning perspective, the contact angle is not the only significant parameter for defining hydrophobicity. For self-cleaning surfaces, a low level of water drop adhesion to the surface is also important. This is the product of the WCA and the contact angle hysteresis (CAH), the difference between advancing and receding contact angles. A combination of high WCA and low CAH results in a decreased force being required to set a droplet in motion [74]. Δθ is small on a chemically homogeneous and hydrophobic surface, this means that a liquid droplet will be unstable and will slide off the substrate if the substrate is tilted (conversely, if the surface chemistry is non-homogenous Δθ will be large and the droplet will be effectively “pinned” to the substrate’s surface.

### 2.4. Hydrodynamics

Hydrodynamic stresses play an essential role in most if not all physiological processes. In particular, cellular processes (i.e., cell morphology, intracellular processes, kinetics and cell to cell signaling) can be easily influenced by hydrodynamics [75]. In designing a material with an antifouling (AF) effect, a deeper understanding of the fluid mechanics at play in the micrometer to nanometer scale is essential [75]. The intertidal zone is an area in the marine environment exposed to air at low tide, and covered in seawater at high tide, leading to a huge diversity of plant and marine life [76]. It is an extremely harsh environment where the effect of a number of stresses (i.e., drag, lift acceleration) on plant and animal life are evident. In the intertidal zone, water velocities can reach between 10 and 15 ms^−1^ [77], and storm waves can reach 25 ms^−1^ in addition to accelerations of more than 400 ms^−2^ [78]. Hydrodynamic forces are said to have a huge effect on the ability of fouling organisms to settle. In this area of the marine environment, marine organisms are not prone to fouling, even though they are subjected to the same fouling pressures as found elsewhere. These non-fouling organisms with enhanced surface topography and optimal hydrodynamics offer an excellent opportunity to develop a non-toxic antifouling solution [76].

Reynolds number is defined as “the ratio of inertial forces to viscous forces in fluid flow”. It essentially expresses the influence of size and shape of organisms moving in a fluid [76,79]. Reynolds number can also be an indicator of the scale separation in fluid flow [79]. Microorganisms (i.e., bacteria, plankton, ciliate) experiencing Reynolds numbers of around 10^−5^ are said to be in an environment in which viscous forces dominate over inertial forces. As a result, bacteria, plankton and ciliate function at low Reynolds number [79]. These hydrodynamic interactions significantly influence the ability of an organism to settle on a surface, allowing an organism to identify a suitable surface. In contrast to this, organisms experiencing Reynolds numbers between 10^3^ to 10^9^ are said to be in an environment in which inertial forces dominate over viscous forces. Therefore, larger organisms and underwater surfaces operate at high Reynolds number [79]. One of the challenges in creating an effective antifouling bioinspired solution is adapting systems that are both effective at low Reynolds number (i.e., over small surfaces) as well as systems that are effective at high Reynolds number (i.e., over large surfaces). Table 3 outlines the variation of Reynolds number experienced by marine organisms with respect to speed [76,79].

### 2.5. Surface Topography

Surfaces from marine organisms capable of reducing or preventing biofilm formation are of interest in engineering and materials sciences [80,81,82]. Biomimetic surface modification has been considered in antifouling material development and a number of studies have examined antifouling potential of topographic patterns, textures and roughness scales found on organisms [4,81,83]. Many marine organisms, as a result of living in the ocean (i.e., shark, dolphin and whale), have evolved characteristics which are understood to prevent the attachment of biofouling organisms on their skin. Table 4 summarizes a range of studied marine organisms and their reported surface characteristics which have been tested for antifouling potential.

One of the earliest known studies of marine-inspired biomimetic AF models investigated was that of the sea fan, *Pseudopterogorgia acerosa* (Pallas) [84]. Vrolijk et al. first presented the research in 1990 with the characterization of two gorgonian octocorals, *Pseudopterogorgia americana* (Gmelin) and *Pseudopterogorgia acerosa* (Pallas). The surface of *P. americana* (Gmelin) was generally smooth, with a dense mucus layer, impeding the observation of surface topography. On the other hand, the surface of *P. acerosa* (Pallas) was discovered as having a surface topography consisting of spicules, with mean surface roughness around 2–4 µm. Contact angle measurements completed on the gorgonian species showed low surface energies of 23–27 mN/m, equating to the region of the Baier curve closely linked with minimal bioadhesion [84,85]. Baier observed this phenomenon in 1973, concluding that surfaces with low critical surface tensions (20–30 mN/m) were “minimally bioadhesive” [85]. It was concluded that the gorgonian species, *P. acerosa*, may use this passive AF mechanism against biofilm formation in the marine environment [84].

Marine invertebrates from the phylum *Echinodermata*, including the starfish, sea urchin or sea cucumber are well-documented as being excellent antifouling models as they have been found to remain largely free from epibiont colonization [15,77]. A study by Bers and Wahl, 2004, investigated brittle sea star, *Ophiura texturata* [30]. Replicas were fabricated, using Devcon 2-TON epoxy resin and Coelan resin pigment following exposure to the field for 28 days and examination by weekly cell counting [30]. Characterizations on the surface of *Ophiura texturata* revealed knob-like structures with diameters of around 10 µm. Results of the study concluded that the sessile organism, *Z. commune*, was repelled by the brittle star in week three whereas *Polydora sp.* was attracted to the brittle star during the last week of the study [30]. This study shows promising results for such textures and offers potential for further exploration if the textures can be replicated in a robust fashion. In a study by Guenther et al. (2007) investigating the AF potential of four tropical sea star species, *Linckia laevigata*, *Fromia indica*, *Cryptasterina pentagonia* and *Archaster typicus* [86], field studies revealed that during the dry season, the topographies had no real effect on the general percentage cover and community composition. These tropical stars consist of a unique topography of paxillae ranging in size from 50–379 µm (height), 108–204 µm (diameter) and 17–108 µm (spacing). These features were replicated using epoxy resin, Devcon 2-TON. However, for two of the stars, *Cryptasterina pentagonia* and *Archaster typicus*, surface topography demonstrated a small effect on the fouling community composition and percentage cover [86]. In 2002, Callow et al. highlighted the effect of topography shape and size on fouling composition and cover [31]. It was observed that the microtopographies of the tropical stars provided favorable attachment points for larvae to settle. This offers some explanation into the higher percentage cover of fouling organisms on the stars in comparison to smooth and rough control surfaces [31]. Again, a species worth considering for further investigation.

*Mytilus edulis*, through biofouling-facilitated invasion, is a model organism for comparison of fouling communities across a wide range of geographical locations [30]. Field studies of the blue mussel, *Mytilus edulis*, with micro-ripples of 1–1.5 µm, was investigated in a study by Bers and Wahl, 2004 [30] revealing an initial AF response but lesser effects as time progressed. Replicas of the surface were made using epoxy resin, Devcon 2-TON and colored using Coelan resin pigment, followed by exposure for 28 days in the marine environment. An initial reduction of barnacle settlement was observed in the first week of the study on the replica, however there was little effect on the inhibition of other fouling organisms in the second and third week, with the final week receiving increased barnacle attachment. Another study by Bers et al. in 2006 [87] investigated the microtopography of *M. edulis* and *Perna perna*. It was reported that the mytilid species with intact microtopographies and periostracum were much less fouled in comparison to “roughened anisotropic surfaces” [87]. Following on from this study, in 2010, Bers et al. [88] conducted a global investigation into the fouling defense mechanisms of mytilid shell microtopographies; *M. edulis*, *M. galloprovincialis*, *Perna perna* and *P. viridis*. These four different mytilid shell species were acquired from eight different regions of the world and the microtopographies were replicated with high resolution resin, Devcon 2-TON. While the results of this study showed a deterrence effect on fouling organisms in the early stages of fouling (weeks 3 and 6), the microtopographies failed to present an antifouling effect in the later stages [88]. In 2003, Scardino et al. studied the microtopography and AF properties of the shell of two bivalve mollusk species, *Mytilus galloprovincialis* and *Pinctada imbricata*. The shells were characterized through AFM and SEM, which revealed ridged topography of 1–2 µm distance and a depth of 1.5 µm for *Mytilus galloprovincialis*. *Pinctada imbricata* characterization revealed a non-repeating topographical pattern [89]. Unlike other studies, the natural shells were exposed in field experiments to the marine environment for 14 weeks. Results of this study concluded that bivalve species, *M. galloprovincialis* was rarely fouled—less than 10% fouled across all size classes of the species whilst *P. imbricata* had much higher levels of fouling [89]. Scardino also characterized blue mussel, *Tellina plicanta* in 2006 to reveal a surface topography of around 2–4 µm [32]. Biofouling assessment was completed using a designed 4 h cell settlement assay. It was concluded inhibition of fouling occurs for microfouling species (i.e., diatom) by limiting the number of favorable attachment points using highly ornate surface topography [32]. However, selective laboratory studies have their limitations. A study by Aldred et al. relates adhesion strength to site selection of common macrofouling organisms such as barnacle cypris larvae. In general, these organisms settle on surfaces that contain a large number of attachment points, leading to high attachment strength. This mechanism is similar to the popular Sharklet topography. Using this mechanism, it was reported that fouling can be significantly reduced, using topographies that present the lowest number of attachment points [90]. In 2006, Guenther documented the diversity of fouling organisms on pearl oyster species, *Pinctada fucata*, *Pteria penguin* and *Pteria chinensis*. *P. fucata* lacked regular microtopography and no significant difference in the diversity of fouling species was observed after 12 weeks and during the 16-week sampling period. However, the fouling communities found on pearl oyster species *Pteria penguin* and *Pteria chinensis* showed significant differences during this time [91]. A study on bivalve species’, *Dosinia japonica* and *Mimachlamys nobilis* was carried out in 2013 [92]. Replication of the texture, carried out in E44 epoxy resin and polyurethane (PU), revealed that topography can prevent the adhesion of *N. closterium* cells with E44 epoxy resin replicated *D. japonica* displaying the best antifouling capability [92].

Replication of a texture in elastomer, poly(dimethyl)siloxane (PDMS) was conducted by Wang et al. who took inspiration from the scales of the yellowfin leatherjacket, *Triacanthus blochii*. A soft lithography process, called nanocasting, was used to replicate the surface microtopography topographical features of the scales consisting of needle-like patterns of height, 300 µm, spacing, 100 µm and diameters between 10–40 µm. Although there were no biofouling experiments carried out during this study, this is the first reported case of fish scale replication and serves as an example of creating highly accurate surface replications, without the added cost [93]. This replication procedure is widely used in areas of study such as microfluidics, and it shows potential, also, for replication of test structures that can be field tested due to the robustness of the material. A recent study carried out by Richards et al. reported the replication of the brill fish, *Scophthalmus rhombus* using a 3-D printing process (Nanoscribe 2-Photon 3-D printing) and soft lithography. The microtexture consisted of micro-ridge features of height, 75 µm, spacing, 10–7 µm and diameter, 10 µm. It was observed that cells attached in greater numbers to the microtexture’s replicated using soft lithography methods (PDMS casting), however further exploration is required. This is the first reported replication of the scales of the brill fish, *Scophthalmus rhombus* [61].

Of the lesser groups of organism’s studies, Crustaceans (or “crustacea”) come from a large family of both marine and land arthropods. This family is defined by the presence of a hard shell or exoskeleton. The colonization of the crab shell surface presents huge advantages for epibionts as the activities of the host (i.e., movement, feeding) may result in feeding chances for other species. However, epibiotic colonization can also cause huge problems in terms of affecting the host’s survival. As a result of this, crustaceans have evolved characteristics to prevent their colonization by epibionts (i.e., burrowing, cleaning) [30,81]. Sullivan et al. investigated the carapace of the crustacean, *Cancer pagurus* for its potential as an antifouling surface. The study reported the chemical composition, spatial distribution, size and shape descriptors of the microscale surface features of *C. pagurus* for the first time [81].

Reported studies that are very promising for marine application suggest that dogfish egg cases have the ability to resist macrofouling up to 6 months (although microfouling still occurs). The dogfish egg case, *Scyliorhinus canicula*, although non-living, provides another biomimetic model for antifouling technologies. On examination of the micro-topographical features present on the surface, ridge-like patterns were revealed of dimensions between 30–50 µm, with a mean surface roughness of approximately 3.7 µm. From this work, surface topography seemed to play a role in the deterring of marine foulers, with most other reported AF surfaces having similar dimension and roughness [97]. Bers also investigated this phenomenon. High resolution replications were made using Devcon 2-TON epoxy resin and colored using Coelan resin pigment. Experiments were conducted in the field for 28 days in the Western Baltic, followed by weekly cell counts and statistical analysis using one-way analysis of variance (ANOVA) and Levene’s Test. Results of this study indicated deterrent effects on microfoulers and initial barnacle reduction [30]. The reproduction of the dogfish egg case, *Scyliorhinus canicula*, paved the way for the development of foul-release (FR) bivalves, providing a first report on the bioinspired production of “natural AF surface” [90].

Shark skin is perhaps the most widely reported bioinspired texture, which has unique topographical patterns with specific characteristics to help the shark in its natural habitat [98,99,100]. The skin has microscale sized ridges which are said to prevent the reproduction of eddies in the turbulent boundary layer (i.e., reducing drag) [76]. The shark skin diamond shaped topography has been found to prevent microorganisms from attaching to the surface. It was also found successful in reducing *Escherichia coli* and *Staphylococcus A* proliferation in hospitals [83].

Other reported bioinspired textures are that of dolphin and whale skin. Early work in this area took place in 1983, by Baier et al. who investigated the antifouling potential of the skin of porpoises and killer whales [90,98]. Characterization of the bottlenose dolphin, *Tursiops truncatus* and killer whale, *Orcinus orca*, exposed micro-topographical ridge features, 0.41–2.35 mm (width) and 7–114 mm (height), reducing drag and fouling significantly. Surface tension measurements were observed to fall within the range for minimal bioadhesion (20–30 mN/m) [90]. The reproduction of porpoise skin, *Tursiops truncatus*, was attempted using tethered polymer chains however the antifouling potential of these replications were never documented [98]. Many cetaceans remain relatively free from fouling organisms, presenting a very clean skin surface. This realization was investigated in 2002, by Baum et al. with the study of pilot whale skin, *Globicephala melas*. The epibiont-free surface of *Globicephala melas* can be credited to the topographical structures on the skin with an average pore size of 0.20 µm^2^. It has been suggested that organisms larger than the scale of microtopography present on the surface will not settle due to reduced attachment points [32]. In this case, the pores present on the surface of *Globicephala melas* were significantly smaller than those of fouling organisms present in the marine environment. This property, along with the speed of the marine mammal (i.e., jumping, surfacing) allows for the removal of weakly bound epibionts, providing fewer favorable attachment points—this has paved the way for the development of nano-structured and nano-pored surfaces [90]. Cao investigated a range of nano-structured and nano-pored surfaces for their AF potential [99]. The surface morphology of *G. melas* was essentially copied using “spray coated multi-layered polyelectrolytes”. This topography succeeded in the prevention of zoospore species, *Ulva*, from settling on the surface—mainly due to the microtopography (600 nm) of the surface under flow. It was also noted that the skin of the pilot whale was in fact, an amphiphilic surface—one which contains dual-wettability, both polar and nonpolar groups [99]. It was reported that the response to morphology was retained, irrespective of chemistry. The lowest level of settlement was observed for structures of the order of 2 mm. The strength of adhesion of settled spores was found to be lowest on the surface with the sub-micrometer-sized features. The authors recommended that in attempts to explore the potential of morphology to deter settlement, hierarchical surfaces are needed to deal with the different preferences of the target organisms. From the wide range of surface textures developed and shown in Table 4, many have been tested in the field.

## 3. Conclusions

This review shows that inspiration from marine organisms has provided surface textures that have been replicated using a variety of fabrication techniques. These textures have been tested in the lab and field for their antifouling potential, with varied success. Many biofouling studies are lab-based using testing with single-celled organisms which are easier to statistically analyze and quantify. However, the success of these AF technologies is likely to be very different when applied under environmental and field conditions.

Of the surveyed papers, many of the marine organism texture features are in the micron range. These vary from 1–10 µm, 100–500 µm with few studies in the nanometer range. Few innovative techniques have been adopted for replication of surface features. The challenge is in meeting the required dimensions as these are limited by the capability of the replication technique, and also the ease of replicating from a small-scale surface to a larger scale. This has been shown to be a challenge using the techniques described in Table 1. However, innovations in roll-to-roll manufacturing can potentially realize the delivery of larger scale replicas of the structure. Micro-contact printing for example or 3-D printing are offering greater flexibility in material development. Although marine inspired surface texture and topography was the focus of this review, an effective AF solution will need to consider combining both surface chemistry, like the very elegant technique by Rosenhahn [100] with suitable topography. While textured surfaces alone have not demonstrated complete antifouling success, evidence suggests that texture plays a significant role.

Existing studies discussed in this review, principally focus on the applicability of the topographies inspired by shark, dolphin and crustacean, for example. However, there are very few novel biomimetic natural surfaces that have demonstrated significant antifouling potential. These textures typically are very complex with hierarchical structures—varying in dimensions. Development and evaluation of fabrication methods to create or replicate patterned surfaces at both micro- and nano-scale levels is required. The replication of effective surface topographies for large scale applications remains a challenge and choice of texture is critical in achieving success. Further research on marine inspired textures with potential antifouling capability, is required to understand the mechanisms involved and the potential for larger scale application.

## Figures and Tables

**Figure 2:**
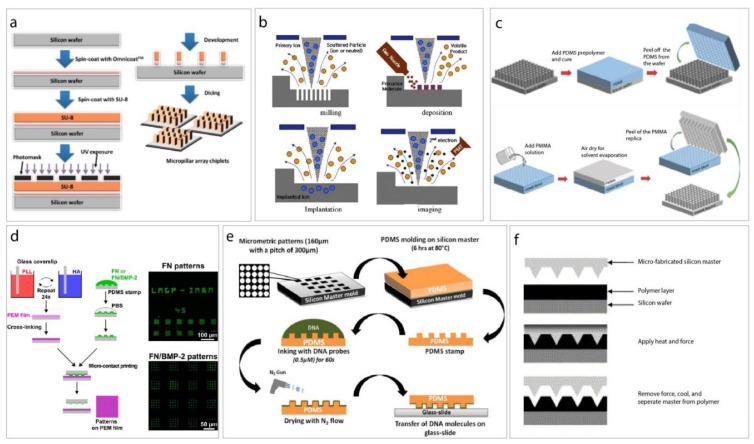
Schematic illustrations of micro- and nano-fabrication methods. (**a**) production of micro-scale surface topographies using photolithography. (**b**) electron beam lithography. (**c**) solvent casting (i.e., soft lithography). (**d**) micro-contact printing. (**e**) direct microcontact printing. (**f**) hot embossing. (available from keaipublishing under open access journal “Bioactive Materials 3” [38]).

**Table 1:** Summary of manufacturing methods commonly used for the production of nano- and micro-scale textured surfaces.

Method	Description	References
Photolithography *	Formation of a pattern in a layer of photoresist which can be transferred by etching into an underlying film (Figure 2a).	[38,39,40]
Electron beam lithography *	Produces surface patterning between 3–5 nm following exposure to electron beam (Figure 2b).	[41,42,43]
Ion beam lithography *	Produces surface patterning of <100 nm due to the nature of the ion.	[41,44,45]
Proximity rolling-exposure lithography (PREL) and electrochemical micromachining (EMM) *	Produces surface patterning over a large surface area, with the ability to produce texturing of various shapes that are otherwise impossible with some of the other techniques.	[46,47,48,49]
Two-photon lithography and atomic layer deposition (ALD) *	Two-photon lithography produces 3-D complex surface topographies with resolutions of around 150 nm, however, requires a photosensitive polymer resin, preventing its use with metallic materials. ALD produces accurate uniform films, offering controllability at atomic level, wafer-scale substrates and high-aspect ratio models. The combination of the two offer a promising tribological solution in small-scale systems.	[50,51]
Soft lithography	Produces topographies at the micro- and nano-scale, using PDMS as a master template (Figure 2c).	[38,52,53,54]
Micro-contact printing *	Involves the fabrication of a “stamp” from PDMS by replica molding, the stamp is covered in ink, pressed and the solvent is left to evaporate, leaving the molecules to be transferred on to the substrate (Figure 2d,e).	[38,55,56]
Hot embossing *	Involves the use of thermoplastic polymers to create micro-patterned surfaces, involving softening the polymer, pressing the template onto the warm polymer and revealing the micro-patterned surface after cooling (Figure 2f).	[38,57,58,59]
3-D printing *	A relatively new technique offering low-cost, efficiency and fast prototyping—requires more in-depth examination.	[38,41,60,61]
Picosecond laser texturing *	Involves the texturing of stainless steel to create an AF superhydrophobic surface. Results indicated a 50% decrease in the mean microbial attachment area ratio—a significant effect in comparison to the untextured stainless steel.	[62,63]

**Note:** The manufacturing methods denoted with an * are commercially available.

**Table 2:** Description of the different scale length topographies observed in common antifouling materials [65].

Scale	Description
Macrotopography; Ra > 10 µm	Surface finishes from cutting tools (i.e., grinding, turning or milling).
Microtopography; Ra ~1 µm	Important in hygienic surfaces.
Nanotopography; Ra < 1 µm	A shiny surface that appears smooth to the eye yet retains nanoscale features on the surface.
Angstrom-scale topography; 1–10 nm	Functional groups on the surface affecting the ability of a cell to sense the surface (i.e., polymer brushes, self-assembled monolayers (SAMs).
Molecular topography; molecules	Influential in surface charge and affects cell-surface binding.

**Table 3:** The variation of Reynolds number in marine organisms with respect to speed.

Reynolds Number	Speed (Approx. ms^−1^)	Organism
10^−5^–10^1^	10^−5^–10^−3^	Bacteria, plankton, ciliate
10	10^−3^–10^−1^	Small fish
10^3^	10^−3^–10^−1^	Large fish
10^5^–10^7^	10^−1^–10	Human swimwear, large fish
10^7^–10^9^	10^−1^–10	Blue whale, large ships

**Table 4:** Summary of bioinspired micro-topographies reported from marine organisms in this review.

Species	Type of Study	Performance	Visual	Reference
Sea fan: *Pseudopterogorgia acerosa* Dimension: Spicules, 2–4 µm	Characterization	Antifouling effect: “Release of fouling” at an ideal surface energy range of 20–30 dyn cm^−1^. AF mechanism: Surface chemistry.	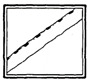	[84]
Brittle star: *Ophiura texturata* Dimension: Knobs, 10 µm in diameter	Field	Antifouling effect: Deterrent effects on microfoulers. AF mechanism: Surface topography.	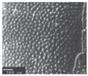	[30]
Sea star: *Linckia laevigata* Dimension: Paxillae 100µm (h), 116 µm (d), 17 µm (spacing)	Field	Antifouling effect: No effect on the fouling composition, community and percentage cover during dry season. AF mechanism: Surface topography.	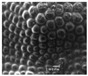	[86]
Sea star: *Fromia indica* Dimension: Paxillae 52 µm (h), 172 µm (d), 108 µm (spacing)	Field	Antifouling effect: No effect on the fouling composition, community and percentage cover during dry season. AF mechanism: Surface topography (requires a combination of behavioral, mechanical and/or chemical antifouling mechanisms).	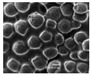	[86]
Sea star: *Cryptasterina pentagonia* Dimension: Paxillae 50 µm (h), 108 µm (d), 103 µm (spacing)	Field	Antifouling effect: No effect during the dry season. Transitory effects on the fouling community composition during wet season. AF mechanism: Surface topography (requires a combination of behavioral, mechanical and/or chemical antifouling mechanisms).	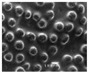	[86]
Sea star: *Archaster typicus* Dimension: Paxillae 379 µm (h), 204 µm (d), 98 µm (spacing)	Field	Antifouling effect: No effect during the dry season. Transitory effects on the fouling community composition during wet season. AF mechanism: Surface topography (requires a combination of behavioral, mechanical and/or chemical antifouling mechanisms).	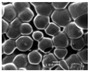	[86]
Mussel: *Perna perna* Dimension: Ripples, 1.5–2 µm	Field	Antifouling effect: Replicas with intact isotropic topographies and smooth controls were much less fouled than roughened anisotropic surfaces [87]. Some deterrent effects observed in weeks 3 and 6. However, the microtopographies were not able to prevent fouling in later stages [88]. AF mechanism: Surface chemistry and topography.	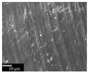	[87,88]
Blue mussel: *Mytilus edulis* Dimension: Micro-ripples, 1–1.5 µm	Field	Antifouling effect: Initial reduction of barnacle settlement [30]. Some deterrent effects observed in weeks 3 and 6. However, the microtopographies were not able to prevent fouling in later stages [88]. AF mechanism: Surface topography.	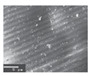	[30,88]
Blue mussel: *Mytilus galloprovincialis* Dimension: Ridges, 1–2 µm (width) and 1.5 µm (depth)	Field	Antifouling effect: Less than 10% across all size classes were fouled [89]. Some deterrent effects observed in weeks 3 and 6. However, the microtopographies were not able to prevent fouling in later stages [88] AF mechanism: Surface chemistry, microtopography and Attachment Point Theory.	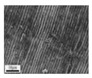	[88,89]
Pearl oyster: *Pinctada impricata* Dimension: Non-repeating pattern	Field	Antifouling effect: High levels of fouling. AF mechanism: Surface chemistry and Attachment Point Theory.	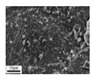	[89]
Bivalve: *Tellina plicanta* Dimension: Projections, 2–4 µm	Laboratory	Antifouling effect: Reduced number of attachment points results in reduced adhesion of diatom species. AF mechanism: Attachment Point Theory.	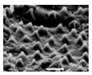	[32]
Mussel: *P. viridis* Dimension: Not disclosed in study	Field	Antifouling effect: Some deterrent effects observed in weeks 3 and 6. However, the microtopographies were not able to prevent fouling in later stages. AF mechanism: Surface topography.	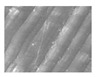	[88]
Bottlenose dolphin: *Tursiops truncatus* Dimension: Ridges, 0.41–2.35 mm (width), 7–114 mm (height)	Laboratory	Antifouling effect: Surface tensions in the range for minimal biofouling attachment (20–30 mN m^−1^), low drag, micro-topographical features contributing to a fouling-free surface. AF mechanism: Surface energy.	−	[90]
Killer whale: *Orcinus orca* Dimension: Ridges, 0.41–2.35 mm (width), 7–114 mm (height)	Laboratory	Antifouling effect: Surface tensions in the range for minimal biofouling attachment (20–30 mN m^−1^), low drag, micro-topographical features contributing to a fouling-free surface. AF mechanism: Surface energy.	−	[90]
Pearl oyster: *Pinctada fucata* Dimension: Non-regular	Field	Antifouling effect: No significant difference in fouling communities after 12 weeks and during the 16-week sampling period. AF mechanism: Combination; physical, chemical and/or environmental.	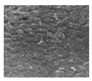	[91]
Pearl oyster: *Pteria penguin* Dimension: Ripples, 0.8 µm	Field	Antifouling effect: Fouling communities found were significantly different both after 12 weeks and during the 16-week sampling period. AF mechanism: Combination; physical, chemical and/or environmental.	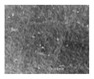	[91]
Pearl oyster: *Pteria chinensis* Dimension: Ripples, 0.6 µm	Field	Antifouling effect: Fouling communities found were significantly different both after 12 weeks and during the 16-week sampling period. AF mechanism: Combination; physical, chemical and/or environmental.	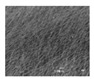	[91]
Bivalve: *Dosinia japonica* Dimension: Ribs, 300–800 nm	Laboratory	Antifouling effect: Topography can prevent the attachment of *N. closterium* cells. AF mechanism: Surface topography.	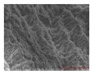	[92]
Bivalve: *Mimachlamys nobilis* Dimension: Pinholes, few microns	Laboratory	Antifouling effect: Topography prone to attachment of *N. closterium* cells. AF mechanism: Surface topography.	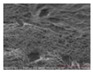	[92]
Yellowfish leatherjacket: *Triacanthus blochii* Dimension: Needles, 100 µm (spacing), 300 µm (height) and 10–40 µm (diameter)	Laboratory	Antifouling effect: First reported replication of *Triacanthus blochii* (yellowfin leatherjacket) using PDMS nanocasting. AF mechanism: Not tested in this study.	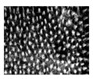	[93]
Brill: *Scophthalmus rhombus* Dimension: Micro-ridges, 74.84 µm (length), 11.7 µm (slope), 16. 6 µm (spacing)	Laboratory	Antifouling effect: First reported replication of *Scophthalmus rhombus* using 3-D printing. AF mechanism: Attachment Point Theory (requires further exploration).	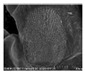	[61]
Crab: *Cancer pagurus* Dimension: Circular elevations, 200 µm and spicules, 2–2.5 µm	Field + Laboratory	Antifouling effect: Repellent to macrofoulers (barnacles) [30]. Settlement of fouling organisms was affected in different ways from the surface microtopographies [81]. AF mechanism: Attachment Point Theory.	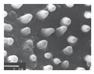	[30,81]
Dogfish egg case: *Scyliorhinus canicula* Dimension: Ridges, 30–50 µm	Field	Antifouling effect: Deterrent effects on microfoulers. Initial reduction of barnacle settlement. No effects of the surface structure of the egg case. AF mechanism: Surface topography.	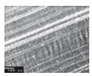	[30]
Shark: Sharklet AF Dimension: Ribs, 2 µm, 2 µm, 4–16 µm (width, spacing, length)	Laboratory (Commercialized)	Antifouling effect: Reduced spore settlement density by 86%. *S. aureus* biofilm percentage cover on Sharklet AF covered surface was 7 % compared to 54 % for smooth PDMS control. AF mechanism: Attachment Point Theory.	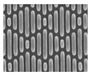	[94,95,96]
Shark: Recessed Sharklet AF Dimension: Ribs, 2 µm, 2 µm, 4–16 µm (width, spacing, length)	Laboratory (Commercialized)	Antifouling effect: *Ulva* spore attachment independent of the area fraction of feature tops and number of features—spores attached in lower numbers here. AF mechanism: Attachment Point Theory.	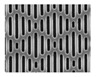	[96]
Shark: Placoid scale Dimension: 2 µm, 1.5 µm, 2 µm (width, height, spacing)	Laboratory	Antifouling effect: Decrease in *E. coli* attachment by 75% when measuring pristine patterns and up to 56% when measuring patterns undergoing extreme mechanical wear. AF mechanism: Attachment Point Theory.	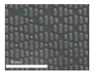	[82]
Pilot whale: *Globicephala melas* Dimension: Ridges, 2 µm and pores, 0.20 µm	Characterization	Antifouling effect: Average pore size (0.20 µm^2^) below that of most biofouling organisms—low numbers of organisms and salt crystals. AF mechanism: Attachment Point Theory.	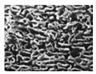	[26]
